# Supplementary material for: Analysis of PPARγ Signaling Activity in Psoriasis
Source: Int J Mol Sci. 2021 Aug 10;22(16):8603. doi: 10.3390/ijms22168603 (PMC8395241; doi:10.3390/ijms22168603)
Supplement: Supplementary file 1 [file ijms-22-08603-s001.zip › Supplemental materials_Analysis of PPARg signaling activity in psoriasis/Pathway models/Models images and html files/Differentiation of psoriatic T cells/44574.html]

IL21


# Protein IL21

|  |  |
| --- | --- |
| URN | urn:agi-llid:59067 |
| Total Entities | 0 |
| Connectivity | 2370 |
| Name | IL21 |
| Description | interleukin 21 |
| Notes | This gene encodes a member of the common-gamma chain family of cytokines with immunoregulatory activity. The encoded protein plays a role in both the innate and adaptive immune responses by inducing the differentiation, proliferation and activity of multiple target cells including macrophages, natural killer cells, B cells and cytotoxic T cells. Dysregulation of this gene plays a role in multiple immune-mediated diseases including lupus, psoriasis and chronic inflammatory diseases. Alternatively spliced transcript variants encoding multiple isoforms have been observed for this gene. [provided by RefSeq, Dec 2011] |
| Primary Cell Localization | Extracellular |
| Class | Ligand |

---

|  |  |
| --- | --- |
| Pathway | Melatonin Effects on Circadian Cycle |
|  | Immunoglobulin Genes Transcriptional Activation |
|  | AHR Signaling in Th17 Cells Function |
|  | Th17-Cell Differentiation |
|  | AHR Signaling in Tr1 Cells Function |
|  | IL6 Expression Targets -> Nociception |
|  | Proteins Involved in Psoriasis |
|  | T-Cells Differentiation Block in Psoriasis |
|  | Proteins Involved in Diabetes Mellitus Type 1 |
|  | Proteins Involved in Celiac Disease |
|  | CD4+T-Cell Response in Celiac Disease |
|  | Proteins with Altered Expression in Celiac Disease |
|  | Celiac Disease |
|  | Th17-Cell Activation in Crohn's Disease |
|  | Proteins Involved in Inflammatory Bowel Diseases |
|  | Proteins Involved in Helicobacter Infections |
|  | Proteins Involved in Diffuse Large-B-Cell Lymphoma |
|  | Follicular Lymphoma |
|  | Hodgkin Lymphoma |
|  | Hodgkin and Reed-Sternberg Cells Reprogramming |
|  | Th17-Cell Differentiation in Asthma |
|  | Th17-Cell and Th1 Immune Responsein Psoriatic Arthritis |
|  | Proteins Involved in Psoriatic Arthritis |
|  | Proteins Involved in Rheumatoid Arthritis |
|  | Th17-Cell Numbers Reduction in HIV |
|  | IL21R -> STAT Signaling |
|  | IL21 Expression Targets |
|  | CD40 -> Expression Targets in Thymus |
|  | TGFB1-TGFBR1/AP-1 Expression Targets |
|  | TGFB1-ACVRL1 Expression Targets |
|  | PPAR Psoriasis |
|  | pprarg neg, uknown targets, ps-positive |
|  | prarg neg,ukn expres targets, ps-positive |
|  | PPARG negative targets |
|  | pparg expr target\_regulators |
|  | Neighbors of disease exacerbation |
|  | Neighbors of tumor progression |
|  | Neighbors of inflammatory status |
|  | Neighbors of overall survival |
|  | Neighbors of disease severity |
|  | Neighbors of tumor size |
|  | Neighbors of mortality |
|  | Neighbors of survival rate |
|  | Neighbors of survival time |
|  | Neighbors of insulin sensitivity |
|  | Neighbors of cancer survival |
|  | Neighbors of graft survival |
|  | Neighbors of therapeutic efficacy |
|  | Neighbors of lethality |
|  | Neighbors of tumor regression |
|  | Neighbors of tumor response |
|  | Neighbors of macrophage count |
|  | Neighbors of long-term survival |
|  | Neighbors of body weight |
|  | Neighbors of neutrophil count |
|  | Neighbors of exhaustion |
|  | Neighbors of apoptosis rate |
|  | Neighbors of viral load |
|  | Neighbors of blood pressure |
|  | Neighbors of vascular leakage |
|  | Neighbors of cancer risk |
|  | Neighbors of cancer incidence |
|  | Neighbors of T-cell count |
|  | Neighbors of antimetastatic activity |
|  | Neighbors of radiosensitivity |
|  | Neighbors of functional status |
|  | Neighbors of pathological response |
|  | Neighbors of lymphocyte count |
|  | Neighbors of disease exacerbation |
|  | Neighbors of tumor progression |
|  | Neighbors of inflammatory status |
|  | Neighbors of overall survival |
|  | Neighbors of disease severity |
|  | Neighbors of tumor size |
|  | Neighbors of mortality |
|  | Neighbors of survival rate |
|  | Neighbors of survival time |
|  | Neighbors of insulin sensitivity |
|  | Neighbors of cancer survival |
|  | Neighbors of graft survival |
|  | Neighbors of therapeutic efficacy |
|  | Neighbors of lethality |
|  | Neighbors of tumor regression |
|  | Neighbors of tumor response |
|  | Neighbors of macrophage count |
|  | Neighbors of long-term survival |
|  | Neighbors of body weight |
|  | Neighbors of neutrophil count |
|  | Neighbors of exhaustion |
|  | Neighbors of apoptosis rate |
|  | Neighbors of viral load |
|  | Neighbors of blood pressure |
|  | Neighbors of vascular leakage |
|  | Neighbors of cancer risk |
|  | Neighbors of cancer incidence |
|  | Neighbors of T-cell count |
|  | Neighbors of antimetastatic activity |
|  | Neighbors of radiosensitivity |
|  | Neighbors of functional status |
|  | Neighbors of pathological response |
|  | Neighbors of lymphocyte count |
|  | Neighbors of cell development |
|  | Neighbors of immune response |
|  | Neighbors of cell population |
|  | Neighbors of T-cell development |
|  | Neighbors of inflammatory response |
|  | Neighbors of angiogenesis |
|  | Neighbors of innate immune response |
|  | Neighbors of cell function |
|  | Neighbors of macrophage activation |
|  | Neighbors of cell formation |
|  | Neighbors of adaptive immune response |
|  | Neighbors of osteoclast development |
|  | Neighbors of epithelial to mesenchymal transition |
|  | Neighbors of immunity |
|  | Neighbors of cell proliferative response |
|  | Neighbors of chemotaxis |
|  | Neighbors of dendritic cell differentiation |
|  | Neighbors of cell survival |
|  | Neighbors of cell phenotype |
|  | Neighbors of T-cell proliferation |
|  | Neighbors of tissue remodeling |
|  | Neighbors of cell adhesion |
|  | Neighbors of T-cell activation |
|  | Neighbors of fibrogenesis |
|  | Neighbors of tumor growth |
|  | Neighbors of dendritic cell development |
|  | Neighbors of cell count |
|  | Neighbors of cell infiltration |
|  | Neighbors of cell interaction |
|  | Neighbors of lumen formation |
|  | Neighbors of cell damage |
|  | Neighbors of monocyte differentiation |
|  | Neighbors of aging |
|  | Neighbors of cell motility |
|  | Neighbors of regeneration |
|  | Neighbors of cell migration |
|  | Neighbors of cancer growth |
|  | Neighbors of phagocytosis |
|  | Neighbors of immune system activation |
|  | Neighbors of leukocyte migration |
|  | Neighbors of osteoclast formation |
|  | Neighbors of cell invasion |
|  | Neighbors of heart function |
|  | Neighbors of hemopoiesis |
|  | Neighbors of ossification |
|  | Neighbors of T-cell function |
|  | Neighbors of immune system function |
|  | Neighbors of autophagy |
|  | Neighbors of senescence |
|  | Neighbors of T-cell response |
|  | Neighbors of tumor immunity |
|  | Neighbors of cell transdifferentiation |
|  | Neighbors of cell growth |
|  | Neighbors of ROS generation |
|  | Neighbors of cancer cell growth |
|  | Neighbors of sensitization |
|  | Neighbors of hemato-encephalic barrier |
|  | Neighbors of macrophage function |
|  | Neighbors of cellular immune response |
|  | Neighbors of colony formation |
|  | Neighbors of macrophage differentiation |
|  | Neighbors of cell differentiation |
|  | Neighbors of keratinocyte proliferation |
|  | Neighbors of life span |
|  | Neighbors of cell homeostasis |
|  | Neighbors of psoriasis |
|  | Neighbors of inflammatory disease |
|  | Neighbors of rheumatoid arthritis |
|  | Neighbors of fibrosis |
|  | Neighbors of colitis |
|  | Neighbors of arthritis |
|  | Neighbors of inflammatory bowel disease |
|  | Neighbors of injury |
|  | Neighbors of metastasis |
|  | Neighbors of carcinogenesis |
|  | Neighbors of multiple sclerosis |
|  | Neighbors of chronic inflammation |
|  | Neighbors of inflammation |
|  | Neighbors of tumor microenvironment |
|  | Neighbors of obesity |
|  | Neighbors of diabetes mellitus |
|  | Neighbors of asthma |
|  | Neighbors of hyperplasia |
|  | Neighbors of liver fibrosis |
|  | Neighbors of leukocyte infiltration |
|  | Neighbors of reperfusion injury |
|  | Neighbors of airway inflammation |
|  | Neighbors of hepatitis |
|  | Neighbors of infection |
|  | Neighbors of preeclampsia |
|  | Neighbors of liver injury |
|  | Neighbors of death |
|  | Neighbors of type 1 diabetes |
|  | Neighbors of encephalomyelitis |
|  | Neighbors of autoimmune disease |
|  | Neighbors of dermatitis |
|  | Neighbors of neutrophil infiltration |
|  | Neighbors of immunopathology |
|  | Neighbors of systemic sclerosis |
|  | Neighbors of hepatocellular carcinoma |
|  | Neighbors of autoimmunity |
|  | Neighbors of gastric cancer |
|  | Neighbors of experimental autoimmune encephalomyelitis |
|  | Neighbors of colorectal cancer |
|  | Neighbors of ischemia |
|  | Neighbors of systemic lupus erythematosus |
|  | Neighbors of melanoma |
|  | Neighbors of collagen-induced arthritis |
|  | Neighbors of cartilage degeneration |
|  | Neighbors of kidney disease |
|  | Neighbors of pulmonary fibrosis |
|  | Neighbors of stroke |
|  | Neighbors of cancer |
|  | Neighbors of neoplasm |
|  | Neighbors of vasculitis |
|  | Neighbors of atrophy |
|  | Neighbors of respiratory hypersensitivity |
|  | Neighbors of synovitis |
|  | Neighbors of endothelial cell dysfunction |
|  | Neighbors of liver disease |
|  | Neighbors of prostate cancer |
|  | Neighbors of ovarian cancer |
|  | Neighbors of intimal hyperplasia |
|  | Neighbors of allograft rejection |
|  | Neighbors of proteinuria |
|  | Neighbors of epithelial cell |
|  | Neighbors of keratinocyte |
|  | Neighbors of monocyte |
|  | Neighbors of macrophage |
|  | Neighbors of adipocyte |
|  | Neighbors of fibroblast |
|  | Neighbors of PBMC |
|  | Neighbors of stromal cell |
|  | Neighbors of microglia |
|  | Neighbors of mononuclear cell |
|  | Neighbors of dendritic cell |
|  | Neighbors of neutrophil |
|  | Neighbors of activated T-cell |
|  | Neighbors of immunocompetent cell |
|  | Neighbors of myeloid cell |
|  | Neighbors of stem cell |
|  | Neighbors of B-cell |
|  | Neighbors of leukocyte |
|  | Neighbors of T-cell |
|  | Neighbors of mast cell |
|  | Neighbors of spleen cell |
|  | Neighbors of hematopoietic cell |
|  | Neighbors of antigen-presenting cell |
|  | Neighbors of bone marrow derived mast cell |
|  | Neighbors of lymphocyte |
|  | Neighbors of Langerhans cell |
|  | Neighbors of lung |
|  | Neighbors of liver |
|  | Neighbors of kidney |
|  | Neighbors of intestine |
|  | Neighbors of lymph node |
|  | Neighbors of colon |
|  | Neighbors of airway |
|  | Neighbors of synovial membrane |
|  | Neighbors of brain |
|  | Neighbors of artery |
|  | Neighbors of lymphatic system |
|  | Neighbors of immune system |
|  | Neighbors of spleen |
|  | Neighbors of eye |
|  | Neighbors of lymph node of mesentery |
|  | Neighbors of thymus gland |
|  | Neighbors of draining lymph node |
|  | Neighbors of mediastinal lymph node group |
|  | Neighbors of NF-kB family |
|  | Neighbors of JNK |
|  | Neighbors of IL1 family |
|  | Neighbors of Jun/Fos |
|  | Neighbors of PI3K |
|  | Neighbors of STAT family |
|  | Neighbors of IFNAR ligand |
|  | Neighbors of transforming growth factor |
|  | Neighbors of cytokine |
|  | Neighbors of interleukin |
|  | Neighbors of ovalbumin |
|  | Neighbors of NF-AT family |
|  | Neighbors of LXR |
|  | Neighbors of IL23 |
|  | Neighbors of T-cell receptor |
|  | Neighbors of IL12 |
|  | Neighbors of mitogen-activated protein kinase kinase |
|  | Neighbors of PP2B |
|  | Neighbors of AHR subfamily |
|  | Neighbors of CD3 complex |
|  | Neighbors of Rho kinase |
|  | comon ps\_pos, pprarg\_neg targets |
|  | Model of PPARG signaling in psoriasis |
|  | PPARG negative regulators and targets |
|  | Model of PPARG related pathways in psoriasis (short version) |
|  | 1\_Differentiation of psoriatic T cells |
|  | 4\_1\_Polymorphisms associated with inflammatory bowel diseases |
|  | 1\_CD4+ T cell and epithelial cell response in celiac disease |
|  | Model of PPARG signaling in psoriais (tested) |
|  | Differentiation of psoriatic T cells |

---

|  |  |
| --- | --- |
| Group | Genes Associated with Systemic Lupus Erythematosus |
|  | Genes Associated with Ulcerative Colitis |
|  | Genes with Mutations Associated with Ulcerative Colitis |
|  | Genes with Mutations Associated with Psoriatic Arthritis |

---

|  |  |
| --- | --- |
| MedScan ID | 59067 |

---

|  |  |
| --- | --- |
| LocusLink ID | 59067 |
|  | 60505 |
|  | 365769 |

---

|  |  |
| --- | --- |
| Alias | interleukin 21 |
|  | interleukin-21 receptor ligand |
|  | DN-452A22.7 |
|  | OTTMUSP00000007838 |
|  | IL21 |
|  | interleukin-21 receptor ligand activity |
|  | Interleukin IL21 |
|  | interleukin-21 receptor ligands |
|  | LOC547223 |
|  | CVID-11 |
|  | Za11 |
|  | LOC194131 |
|  | CVID11 |
|  | IL-21 |
|  | interleukin-21 isoform |
|  | interleukin-21 |

---

|  |  |
| --- | --- |
| GO ID | 0005125 |
|  | 0005126 |
|  | 0005134 |
|  | 0048469 |
|  | 0006955 |
|  | 0038114 |
|  | 0030890 |
|  | 0042102 |
|  | 0008284 |
|  | 0001819 |
|  | 0050729 |
|  | 0045078 |
|  | 0032740 |
|  | 0045954 |
|  | 0034105 |
|  | 0042531 |
|  | 0007165 |
|  | 0007260 |
|  | 0005576 |
|  | 0005615 |
|  | 0001783 |
|  | 0045579 |
|  | 0032733 |
|  | 0002729 |
|  | 0032825 |
|  | 0010469 |
|  | 0005622 |
|  | 0042511 |
|  | 0042517 |
|  | 0042503 |

---

|  |  |
| --- | --- |
| KEGG ID | hsa:59067 |
|  | mmu:60505 |
|  | rno:365769 |

---

|  |  |
| --- | --- |
| Organism | Homo sapiens {Organism urn:agi-taxid:9606} |
|  | Mus musculus {Organism urn:agi-taxid:10090} |
|  | Rattus norvegicus {Organism urn:agi-taxid:10116} |
|  | Mus musculus |
|  | Homo sapiens |
|  | Rattus norvegicus |

---

|  |  |
| --- | --- |
| Mouse chromosome position | 3 |

---

|  |  |
| --- | --- |
| OMIM ID | 605384 |
|  | 615767 |

---

|  |  |
| --- | --- |
| Rat chromosome position | 2q25 |

---

|  |  |
| --- | --- |
| Hugo ID | 6005 |
|  | HGNC:6005 |

---

|  |  |
| --- | --- |
| Human chromosome position | 4q27 |
|  | 4q26-q27 |

---

|  |  |
| --- | --- |
| Swiss-Prot Accession | Q9HBE4 |
|  | A0A224B028 |
|  | Q9HBE4.3 |
|  | E9PX58 |
|  | Q5SUE2 |
|  | Q9ES17 |
|  | Q9ES17.1 |
|  | A3QPB9 |
|  | A3QPB9.1 |
|  | A5J0L4 |
|  | Q9HBE4.2 |

---

|  |  |
| --- | --- |
| GenBank ID | NC\_000004 |
|  | NM\_021803 |
|  | NP\_068575 |
|  | NM\_001207006 |
|  | NP\_001193935 |
|  | NG\_031966 |
|  | AC053545 |
|  | AY763518 |
|  | AAU88182 |
|  | CH471056 |
|  | EAX05226 |
|  | CS080568 |
|  | CAI94500 |
|  | CS237090 |
|  | CAJ47524 |
|  | CS450761 |
|  | CAL81203 |
|  | CS582814 |
|  | CAN87399 |
|  | FB677173 |
|  | CAS03522 |
|  | GM619752 |
|  | CAV33288 |
|  | HB976773 |
|  | CBE74752 |
|  | HC196689 |
|  | CBI70418 |
|  | HC203173 |
|  | CBI85469 |
|  | HC203434 |
|  | CBI85472 |
|  | HC686913 |
|  | CBL93962 |
|  | JA104585 |
|  | CCA63962 |
|  | AF254069 |
|  | AAG29348 |
|  | BC066258 |
|  | AAH66258 |
|  | BC066259 |
|  | AAH66259 |
|  | BC066260 |
|  | AAH66260 |
|  | BC066261 |
|  | AAH66261 |
|  | BC066262 |
|  | AAH66262 |
|  | BC069124 |
|  | AAH69124 |
|  | CD559460 |
|  | CD559613 |
|  | DQ645417 |
|  | ABG36529 |
|  | LC133256 |
|  | BBA22643 |
|  | Q9HBE4 |
|  | NC\_000069 |
|  | NM\_021782 |
|  | NP\_068554 |
|  | NM\_001291041 |
|  | NP\_001277970 |
|  | NT\_039248 |
|  | NT\_039252 |
|  | AL645807 |
|  | AL645966 |
|  | AL645982 |
|  | AL662823 |
|  | CH466530 |
|  | EDL35100 |
|  | AF254070 |
|  | AAG29349 |
|  | AK155984 |
|  | AY428162 |
|  | AAR06254 |
|  | BC125414 |
|  | AAI25415 |
|  | BC125416 |
|  | AAI25417 |
|  | DQ645418 |
|  | ABG36530 |
|  | Q9ES17 |
|  | NC\_005101 |
|  | NM\_001108943 |
|  | NP\_001102413 |
|  | AC\_000070 |
|  | AABR07010109 |
|  | AAHX01016352 |
|  | AAHX01016353 |
|  | CH473961 |
|  | EDM01297 |
|  | HB976777 |
|  | CBE74756 |
|  | DQ387062 |
|  | ABD52001 |
|  | A3QPB9 |
|  | NC\_018915 |
|  | AMYH02010018 |
|  | HQ447458 |
|  | ADQ31944 |
|  | AC\_000025 |
|  | AAHY01025628 |
|  | AC\_000136 |
|  | ABBA01044738 |
|  | ABBA01044739 |
|  | XM\_006535513 |
|  | XP\_006535576 |
|  | CAM28076 |
|  | CAI26234 |
|  | CAM18421 |
|  | NT\_016354 |
|  | NW\_922217 |
|  | NW\_001838920 |
|  | AC\_000047 |
|  | NT\_162143 |
|  | NW\_001030719 |
|  | Q5SUE2 |
|  | NW\_047625 |
|  | NW\_001084800 |
|  | XM\_001479075 |
|  | XP\_001479125 |
|  | XM\_622579 |
|  | XP\_622579 |

---

|  |  |
| --- | --- |
| Swiss-Prot ID | IL21\_HUMAN |
|  | IL21\_MOUSE |
|  | IL21\_RAT |
|  | Q5SUE2\_MOUSE |

---

|  |  |
| --- | --- |
| Cell Localization | Secreted |
|  | Extracellular |

---

|  |  |
| --- | --- |
| Ensembl ID | ENSG00000138684 |
|  | ENSP00000477555.1 |
|  | ENST00000611104.2 |
|  | ENSP00000497915.1 |
|  | ENST00000648588.1 |
|  | ENSMUSG00000027718 |
|  | ENSMUSP00000124668.1 |
|  | ENSMUST00000161015.1 |
|  | ENSMUSP00000029273.1 |
|  | ENSMUST00000029273.7 |
|  | ENSRNOG00000017376 |
|  | ENSRNOP00000023348.4 |
|  | ENSRNOT00000023348.4 |
|  | ENST00000264497.7 |
|  | ENSP00000264497.3 |
|  | ENST00000611104.1 |
|  | ENSP00000477555 |
|  | ENST00000611104 |
|  | ENSP00000264497 |
|  | ENST00000264497 |
|  | ENSMUSP00000124668 |
|  | ENSMUST00000161015 |
|  | ENSMUSP00000029273 |
|  | ENSMUST00000029273 |
|  | ENSRNOP00000023348 |
|  | ENSRNOT00000023348 |

---

|  |  |
| --- | --- |
| MGI ID | MGI:1890474 |
|  | 1890474 |

---

|  |  |
| --- | --- |
| RGD ID | 1307384 |

---

|  |  |
| --- | --- |
| Unigene ID | Rn.214949 |
|  | Mm.157689 |
|  | Mm.393490 |
|  | Hs.567559 |

---

|  |  |
| --- | --- |
| Homologene ID | 11032 |

---

|  |  |
| --- | --- |
| Shape | Rhomb |

---

|  |  |
| --- | --- |
| IPI ID | IPI00029151 |
|  | IPI00877605 |
|  | IPI00368229 |
|  | IPI00112642 |

---
